# Sensitivity as outcome measure of androgen replacement: the AMS scale

**DOI:** 10.1186/1477-7525-4-23

**Published:** 2006-03-30

**Authors:** Lothar A Heinemann, Claudia Moore, Juergen C Dinger, Diana Stoehr

**Affiliations:** 1 Center for Epidemiology & Health Research Berlin, Invalidenstr. 115, 10115 Berlin, Germany; 2 Jenapharm, Medical Affairs Andrology, Otto-Schott-Str. 15, 07745 Jena, Germany; 3 University of Wuerzburg, Chair of Statistics, Am Hubland, 97074 Würzburg, Germany

## Abstract

**Background:**

The capacity of the AMS scale as clinical utility and as outcome measure still needs validation.

**Methods:**

An open post-marketing study was performed by office-based physicians in Germany in 2004. We analysed data of 1670 androgen-deficient males who were treated with testosterone gel. The AMS scale was applied prior to and after 3 months treatment.

**Results:**

The improvement of complaints under treatment relative to the baseline score was 30.7% (total score), 27.3% (psychological domain), 30.5% (somatic domain), and 30.7% (sexual domain), respectively. Patients with little or no symptoms before therapy improved by 9%, those with mild complaints at entry by 24%, with moderate by 32%, and with severe symptoms by 39% – compared with the baseline score. We showed that the distribution of complaints of testosterone deficient men before therapy almost returned to norm values after 12 weeks of testosterone treatment. Age, BMI, and total testosterone level at baseline did not modify the positive effect of androgen therapy. We also demonstrated that the AMS results can predict the independent (physician's) opinion about the individual treatment effect. Both, sensitivity (correct prediction of a positive assessment by the physician) and specificity (correct prediction of a negative assessment by the physician) were over 70%, if about 22% improvement of the AMS total score was used as cut-off point.

**Conclusion:**

The AMS scale showed a convincing ability to measure treatment effects on quality of life across the full range of severity of complaints. Effect modification by other variables at baseline was not observed. In addition, results of the scale can predict the subjective clinical expert opinion on the treatment efficiency.

## Background

The Aging Males' Symptoms (AMS) scale was originally developed in Germany as a health-related quality of life scale (HRQoL) [1]. The scale was designed as self-administered scale (a) to assess symptoms of aging (independent from those which are disease-related) between groups of males under different conditions, (b) to evaluate the severity of symptoms over time, and (c) to measure changes pre- and post androgen therapy [1]. It was developed in response to the lack of fully standardized scales to measure the severity of aging symptoms and their impact on HRQoL in males, specifically [2,3]. It was recently demonstrated by a French research group that the AMS scale measures HRQoL similarly in younger (even 20–30 years old) and older persons [4]. Right from the beginning a possible screening potential of the AMS scale was controversially discussed. Therefore we compared the AMS scale with internationally well-known screening instruments for androgen deficiency in adult males (ADAM scale of Morley et al [5] and the Screener of Smith' et al [6]. We found that the AMS has obviously similar test characteristics as both screening instruments [7]. Later, the similarity of AMS and ADAM was confirmed in another study [8]. In addition, Kratzik et al [9] observed in a population-based cross-sectional study in Vienna an impressive association between subscales of the AMS and free testosterone level when age and body mass index was multivariately taken into account. Recently, a Japanese research group under Itoh et al [10] and Soh et al [11] observed a correlation between the AMS scores and the testosterone level. A Polish research group found a similar but less clear result [12]. Other studies, however, could not find associations of the AMS scores with testosterone level [13,14].

Meanwhile, the AMS scale was internationally well accepted: it is now available in 21 languages [2,15,16], and can be down loaded from the internet .

The evaluation of the AMS scale is simple; the scheme has been published [4]. Norm values to compare with were determined [1,3]. Conventional psychometric requirements of test reliability and validity have been successfully achieved and published [17,18]. A point was reached to demonstrate the capacity of the scale to reliably measure the effect of androgen treatment or to predict the magnitude of the therapeutic effect subjectively perceived by the treating physician. To this end, many clinicians use the term "validity" and mean high utility for clinical work or research.

It was shown in a previous publication that the AMS scale meets the requirements of a clinical utility and outcomes sensitivity [7]. The focus of this paper however is to analyze if variables such as age, body mass index (BMI), severity of complaints, and testosterone level before treatment effect the outcome measured with the AMS scale.

## Methods

An open post-marketing study was conducted together with office-based physicians in Germany in 2004. The study monitored the effect of androgen substitution on complaints as well as adverse reactions of a licensed testosterone gel product (Testogel JENAPHARM^®^) under routine conditions. The eligibility of male patients for androgen therapy was determined by the prescribing physician, i.e. following the recommendations of the International Society for The Study of the Aging Male (ISSAM) [19] for testosterone treatment in patients with androgen deficiency. No other inclusion or exclusion criteria were set up except the consent between the patient and his treating doctor. The needed sample size was not determined for this observational three months follow-up study.

The observation encompassed three visits which were documented in a short form: before treatment, after 4–6 weeks, and at the end of the observation period of 12 weeks. A short questionnaire was completed by the treating physician to characterize the patient at baseline, after 4–6 weeks androgen treatment, and at the end of the study (3 months). The physician subjectively assessed the treatment effect at each of the three visits, and listed also adverse reactions on a specific form.

We got for this methodological paper a database with the AMS scale completed before therapy and after 12 weeks, age, BMI and total testosterone (TT). However, only a sub-sample had TT values available.

The computerized data of this post-marketing study were analyzed with conventional statistics using the statistical package SAS 9.1^®^.

## Results and discussion

Altogether, 1670 patients were available for analysis. A general description of the group analyzed in this paper is given in Table 1. The mean age was of 56.4 years, the mean BMI was 26.8, and the TT at baseline was 2.5 ng/ml. A great proportion of the participants had a medical history of one or more chronic diseases. In addition, alcohol consumption and smoking were quite frequent (see table 1).

**Table 1:** Baseline parameters of the study participants

	n^a^	Mean (S.D)
Age (years)	1670	56.4 (10.8)
Body mass index (kg/m^2^)	1670	26.8 (3.1)
Testosterone-level at baseline (ng/ml)	1670	2.5 (1.1)
	n^b^	%

Smoker: Yes, current smoker	1661	32.1
Alcohol (yes: often/regularly)	1660	11.9
Diabetes mellitus (yes)	1542	16.9
Hypertension (yes)	1578	34.2
Cardiovascular conditions (yes)	1533	12.0
Chronic pulmonary conditions (yes)	1504	8.1
Tumour (yes)	1527	9.0

n^a ^number of men who had no missings in certain variables which were used as independent variables for analysis; n^b ^proportion of the total of 1670 men who provided information on a certain parameter.

The HRQoL improved after 12 weeks of testosterone-gel application as measured with the total score of the AMS scale. Relative to the scores at baseline, the total score, the scores for the psychological, somato-vegetative, and sexual scores improved 30.7%, 27.3%, 30.5%, and 30.7% compared with the baseline score, respectively. This is an almost identical relative improvement of the HRQoL as shown in an earlier study associated with injectable testosterone [7].

**Figure 1:**
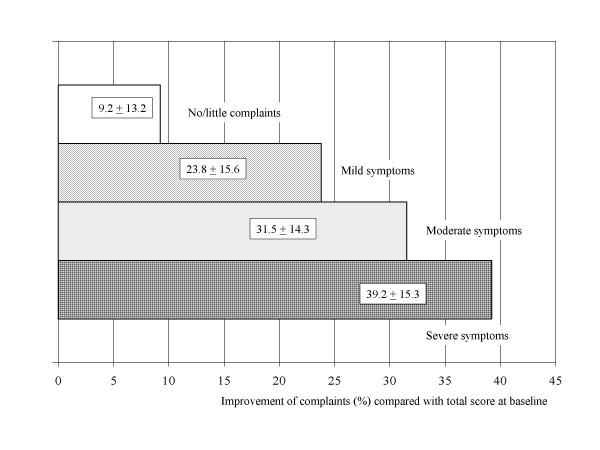
Improvement of complaints under androgen therapy. Difference between pre- and post-treatment AMS total score divided by pre-treatment score in percent (%). Stratification by four categories of severity of complaints at baseline.

The higher severity of complaints at baseline the greater is the improvement as demonstrated for the AMS total score in Figure 1. This applies also for the three sub-scales (data not shown). This was expected but an important observation with impact on the methodological assessment (validity) of the AMS scale.

**Figure 2:**
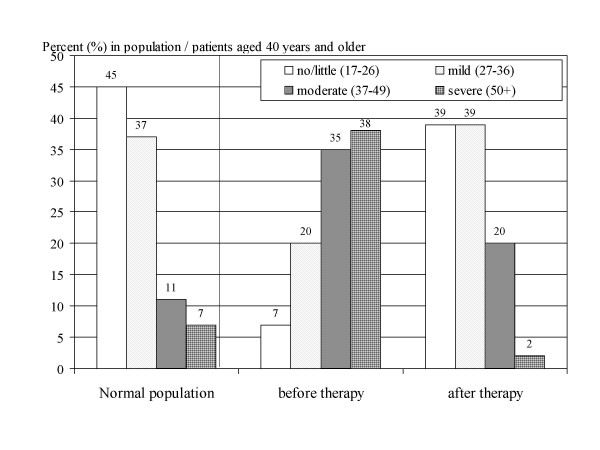
Relative frequency distribution in four categories of severity of complaints measured with AMS (total score): in the normal male population [1, 3] (left side), in patients with AD before and after therapy (middle and right columns).

Compared with the distribution of complaints in the normal population (=norm values [1,3]), the markedly altered HRQoL in this androgen deficient males shifted towards the "norm" of the male population over 40 years after androgen treatment (Figure 2). Patients under 40 were excluded for this comparison (n = 137). It seems to be important to underscore: There was a positive effect (taken numbers at face value) detectable even in males with mild or even little symptoms. Almost identical observations concerning improvement of complaints after androgen therapy were made for injectable testosterone in a previous study [17]. Thus, two independent observational studies (with the same methodology) found the same pattern, but there is still a need for confirmation in a randomised clinical trial.

To answer the question if the treatment-related improvement depends on age, BMI, and testosterone level at baseline, we run a stratified analysis (one-way analysis of variance). Table 2 shows the relative improvement after therapy for the AMS total score, and also for the three sub-scores. There is neither much difference in relative improvement among age, BMI or testosterone groups nor among subscales. All significant changes of HRQoL range around 30% improvement compared with baseline (before therapy). There is no clinically consistent and relevant impact on the magnitude of improvement of the three variables at baseline (age, BMI, TT) considering the size of the standard deviation (in brackets), although the Tuckey-Test showed a few significant effects of age and BMI but with contradictory direction of the trend of the effect, i.e. random findings due to multiple testing cannot be excluded. It cannot be excluded either that the study group is too homogeneous to find small effects.

**Table 2:** Improvement of AMS scores after testosterone-gel therapy. Stratification by age, BMI, and testosterone categories at baseline. The relative improvement is the difference between the pre- and post-treatment score divided by pre-treatment score as percents (%). Wilcoxon signed rank test was used to test differences of significance

		Total score		Psychological score		Somatic score		Sexual score	
	n	%	p	%	P	%	p	%	p
All	1670	30.7 (17.3)	< .0001	27.3 (22.4)	< .0001	30.5 (18.7)	< .0001	30.7 (20.6)	< .0001
Age									
< 50	380	28.0 (18.4)	< .0001	23.8 (22.6)	< .0001	27.4 (20.0)	< .0001	28.3 (23.1)	< .0001
50–59	577	33.2 (17.6)	< .0001	29.4 (22.9)	< .0001	32.3 (19.2)	< .0001	34.4 (20.0)	< .0001
60+	713	30.1 (16.3)	< .0001	27.5 (21.6)	< .0001	30.6 (17.3)	< .0001	29.0 (19.1)	< .0001
BMI (kg/m^2^									
< 24.8	408	28.9 (17.5)	< .0001	25.0 (22.5)	< .0001	28.7 (18.4)	< .0001	28.8 (21.5)	< .0001
24.8 – 28.3	849	32.6 (16.3)	< .0001	29.4 (21.6)	< .0001	32.0 (18.1)	< .0001	33.1 (18.7)	< .0001
28.4+	413	28.4 (18.7)	< .0001	25.2 (23.5)	< .0001	28.9 (19.9)	< .0001	27.5 (22.6)	< .0001
Total testosterone (ng/ml)									
< 1.81	424	31.0 (17.8)	< .0001	27.2 (22.5)	< .0001	31.1 (19.8)	< .0001	30.3 (21.8)	< .0001
1.81 – 2.99	832	31.3 (16.3)	< .0001	28.0 (21.8)	< .0001	30.8 (18.0)	< .0001	31.6 (18.8)	< .0001
3.00+	414	29.1 (18.6)	< .0001	26.1 (23.4)	< .0001	29.1 (18.9)	< .0001	28.2 (23.4)	< .0001

Another question was to what extend the evaluation with the AMS scale could "predict" the opinion of the treating physician regarding "success" of the androgen therapy. The treating physician subjectively assessed the "success" without knowing the results of the AMS he had no access to. Sensitivity (correct prediction of a positive assessment by the physician) and specificity (correct prediction of a negative assessment by the physician) are important characteristics for a test that intends to "diagnose" successful therapy – like AMS in this case (predictive validity).

As can be seen in Table 3, we plotted the sensitivity and specificity in a kind of ROC analysis against the degree of improvement of complaints found under androgen treatment (difference between the pre- and post-treatment score on AMS as percent of pre- treatment total score). More than 22% relative improvement of the total AMS score seems a suitable cut-off point for "diagnosing treatment success": both sensitivity and specificity were about 70% and thereby acceptably high. In other words, the AMS scale is able to assess the treatment success with sufficient good test characteristics, validated against the expert opinion of the treating physician. It could be an advantage of a "success diagnosis" with the AMS scale because it is directly based on patients' view, whereas the physician's assessment could be rather a varying mixture of theoretical expectation/experience and patient's report. Anyway, since the methodological characteristics of the AMS scale are pretty good it is worthwhile to get experience with this tool in clinical practice. It might be recommendable to apply a standardized "objective" scale like the AMS scale in clinical studies, and in addition – if necessary – the subjective, not standardizable judgment of a physician.

**Table 3:** Sensitivity and specificity of potential cut-off points for the "diagnosis of treatment success" using the relative improvement of AMS total score (ROC-Analysis)

**Cut-off Point relative score improvement**	**Sensitivity (%)**	**Specificity (%)**
≥5 %	93.5	24.0
≥10 %	90.7	32.8
≥15 %	84.9	49.6
≥20 %	76.3	64.8
≥22 %	73.6	70.4
≥25 %	67.2	78.4
≥30 %	56.3	87.2
≥35 %	43.9	92.8
≥40 %	31.1	93.6

It seems important to underline that this paper has a sufficient basis to describe the validity of the scale, but there is no intention to discuss the efficacy of testosterone substitution. This would be the task of a double-blinded, placebo controlled trial. We could just demonstrate that the AMS as measure of health-related quality of life (HRQoL) is able to detect changes in quality of life following androgen substitution in androgen-deficient males – irrespective of the specific drug used as treatment or the specific underlying diagnosis in this observational follow up study. This confirms results of an earlier post-marketing study [7] that showed acceptably good validity.

The AMS scale can be applied in clinical trials both in young hypogonadel men as well as in late-onset hypogonadism according to French experience [4]: The scale measures the same phenomenon in different age groups, and no obvious differences exist in reference values. We recommend the use of the total score as outcome measure, but domain scores can be used as well. From our current experience we would conclude that the same cut-off point should be used as "treatment success" when planning a trial. However, we had no access to data from randomized clinical trials to really investigate this issue.

## Conclusion

The AMS scale showed a convincing ability to measure treatment effects on quality of life across the full range of severity of complaints. Effect modification by other variables at baseline (age, BMI, and testosterone level) on the "treatment associated effect on AMS score" was not observed. In addition, results of the scale can predict the subjective clinical expert opinion on the treatment efficiency.

## Competing interests

CM is employee of the company that produces testosterone products. The authors, however, see no conflict of interest as far as the methodological aspects of validation of the AMS scale are concerned.

## Authors' contributions

LAH: developer of the AMS scale, responsible for the design of this validation analysis, and involved in writing the manuscript; CM: responsible for design and execution of the PMS, contributed to writing of the paper; JD: contributed to the design of the study, and to writing the manuscript; DS: responsible for setting up and managing the database for different analyses, running all analyses, and contributes to revising the paper.
